# Reconsidering inherent requirements: a contribution to the debate from the clinical placement experience of a physiotherapy student with vision impairment

**DOI:** 10.1186/s12909-016-0598-0

**Published:** 2016-02-27

**Authors:** Kylie N. Johnston, Shylie Mackintosh, Matthew Alcock, Amy Conlon-Leard, Stephen Manson

**Affiliations:** School of Health Sciences, University of South Australia, GPO Box 2471, Adelaide, South Australia 5001 Australia; Physiotherapy Department, Royal Adelaide Hospital, North Terrace, Adelaide, South Australia 5000 Australia; Learning and Teaching Unit, University of South Australia, GPO Box 2471, Adelaide, South Australia 5001 Australia

**Keywords:** Vision impairment, Inherent requirements, Physiotherapy, Clinical education

## Abstract

**Background:**

Clinical placements in acute hospitals present challenges for students with vision impairment who are being educated as health care professionals. Legislation in Australia supports reasonable adjustments to education, thus students with vision impairment have completed accredited courses and gained professional registration. However the implementation of inherent requirement statements suggesting that adequate visual acuity is required to complete a physiotherapy program may create barriers to access for such students.

**Methods:**

We describe features that contributed to a successful physiotherapy clinical placement in an acute hospital setting for a student with vision impairment and use this experience to prompt debate about the use of inherent requirement statements.

**Findings:**

Planning, consultation, collaboration and problem solving commencing from the time of program entry were integral to clinical placement preparation for this student. Individualised adjustments (including a support worker for reading screens and medical records) and the student’s specific qualities (professionalism, communication, problem solving, memory, kinaesthetic abilities) contributed to a successful outcome.

**Discussion:**

Reflecting on this experience and published inherent requirements, there is an apparent lack of involvement of people with disability in the development of inherent requirement statements; we question the need for this level of regulation; and discuss the potential impact of inherent requirement statements on the health workforce.

**Summary:**

This experience demonstrated that an individualised approach to reasonable adjustments for a student with a disability was successful in an acute hospital setting. The implementation of inherent requirement statements may systemically reduce the capacity of education providers to develop such bespoke solutions and deserves further debate.

## Introduction

While students with disabilities are under-represented in tertiary education [1], support for access to tertiary education for health professional students with disabilities has been addressed in legislation [2, 3]. A tension has emerged between inclusive practices legislated in education and competency-based education and registration for health care professionals [4, 5]. This tension is most obvious in courses where students are experiential learners in clinical placements at health care facilities and practices [4]. Some believe that people with disability are unsuitable for health professional practice. For example a 2004 survey of nursing students, clinical nurses and nursing educators reported evidence of “negative, deficit views of students with disabilities, and even hostility towards the notion of the inclusion of such students”[6].

An approach by universities to exclude students whom they consider inappropriate for their programs has been to develop inherent requirement statements. Inherent requirements seek to define core aspects of the curriculum that must be fulfilled in order for the course to be completed [5, 7, 8]; however there is no international or Australian agreement on their definition, nor are they part of disability legislation. The purpose of this paper is: firstly to report factors that contributed to an effective physiotherapy clinical placement in a hospital setting for an Australian student with vision impairment; and secondly, to discuss this experience as an example of an alternative approach to the policy of development and implementation of inherent requirement statements. If inherent requirements were implemented at the University of South Australia, it is likely that students with vision impairment would be excluded from the physiotherapy programs. In this way we seek to contribute our real world experience and provide a positive example of inclusive practice as an alternative voice in the current debate on this issue.

## Background

Clinical placements are a key component of education to prepare physiotherapy students for professional registration [9]. These placements provide students with opportunities to develop the competencies required for completion of a physiotherapy qualification in an accredited programme in Australia [9]. The interface with patients in the clinical placement setting requires particular attention to ensure this is a successful learning experience for students and a safe, effective interaction for patients.

Students with disabilities have been the fastest growing equity cohort in Australia with enrolments increasing by 58 % during the period 2007–2013 [10]. Australian education providers are legally required to provide protection against discrimination due to a disability and to support people with disabilities to engage in education [3]. This includes the negotiation of support to give physiotherapy students with disabilities fair access to the clinical placement environment, allowing them to develop the required clinical competencies. If a student has a vision impairment, placements in an acute hospital environment present potential challenges for the implementation of the Act as it is a complex, unpredictable, high acuity setting which uses real-time screen-based monitors and paper-based medical records.

In the Australian setting there is a paucity of published information regarding physiotherapists or physiotherapy students with vision impairment. In contrast, physiotherapy is an established, accessible career for people with vision impairment in the UK [11, 12]. The Chartered Society of Physiotherapists has published specific guidance for physiotherapy academic staff and clinical educators working with students with vision impairment [13]. However, reports by students and education providers suggested challenges and barriers remained in the implementation of such guidance. A Delphi investigation of physiotherapy admitting officers in UK physiotherapy programs explored implementation of the 1995 UK Disability Discrimination Act in physiotherapy education [14]. Respondent concerns included the necessary levels of ability, adequacy of support for students with disability and the concept of conditional qualification. Interviews with three physiotherapy students/recent graduates with vision impairment in the UK identified that staff behaviours and resource availability both had potential influence learning experiences. Extra time and effort required to complete course requirements was acknowledged in all cases, and concerns about disclosure were expressed in one case [15]. Clinical placements were not specifically referred to in this study.

Articulation of inherent requirement statements in tertiary education has been proposed in Australian universities to support inclusive curriculum design [7]. Detailed inherent requirements have been defined at least one Australian university offering a physiotherapy program as “the fundamental components of a course or unit, that are essential to demonstrate the capabilities, knowledge and skills to achieve the core learning outcomes of the course or unit, while preserving the academic integrity of the university’s learning, assessment and accreditation process”[8]. With such requirements, it appears that reasonable supports may not be able to be implemented to enable people with disability to participate in these aspects of education as “fulfilment of such requirements is non-negotiable” [7]. Specific inherent requirement statements in relation to the physiotherapy program include a requirement for “adequate visual acuity ….to provide safe and effective physiotherapy management” [16]. Similar statements are described for other Australian health [17, 18] and law degrees [19]. These statements align with a submission to a 2012 review of the Disability Standards for Education [20] which suggested that tertiary institutions should publish inherent requirements for all programmes to “… allow students with a disability to select courses confident that they understand, and can reasonably expect to meet, the inherent requirements of that course” ([20], p72). However, the submission was not adopted by the government response to the review and so has no legal standing.

Employment is an issue for people with a disability. Australia is one of the worst performing countries in the Organisation for Economic Cooperation and Development (OECD) with regard to employment rates for people with disabilities. In 2010 employment rates for people with disability were reported to be 40 % and declining with almost one in two people with disability in Australia living in poverty [21]. Education may be a key to improving employment rates in this sector as Australian graduates with disability have similar employment outcomes to the broader graduate cohort. In 2013, 69.3 % of Australian graduates with disabilities were in full time employment [22]. Consequently, the decision of people with disabilities to seek tertiary qualifications to improve their employment prospects is a positive one. However, the development and publication of statements of inherent requirement by education providers may create unnecessary and potentially illegal barriers to access rather than supporting the rights of people with disabilities in higher education.

This report aims (1) to share key features that contributed to an effective physiotherapy clinical placement in acute hospital setting for an Australian student with vision impairment; and (2) to discuss our experience as an example of an alternative approach to the policy of development and implementation of inherent requirement statements.

## Methods

### Context

Andrew (pseudonym) is legally blind and has 5 % vision. Andrew’s case is unusual in our physiotherapy program, which has educated only two students with significant vision impairment during the last 20 years. His five week, seven hour/day placement was conducted at a metropolitan hospital with a caseload of acute cardiorespiratory and orthopaedic patients in the third year of a four year course of study toward a Bachelor of Physiotherapy degree.

### Evaluation

At the successful completion of Andrew’s clinical placement, we evaluated this teaching and learning experience from the perspective of the student and his clinical educator. Semi structured interviews (Table 1) were conducted by an investigator with (1) the student and (2) the clinical educator. The interviews lasted between 30 min and one hour. Interviews were audiotaped and written notes were taken at the time of interview.

**Table 1:** Semi structured interview questions

1. What made this placement work?	
2. What useful information can we pass on to other physiotherapy courses and clinical educators in the hospital setting who offer placement to a physiotherapy student with vision impairment?	
3. Are there issues that still need to be addressed?	
4. Were the specific supports put in place sufficient?	
5. Did the need for supports change over the placement?	
6. How did you deal with the issue of disclosure?	
7. Were there specific qualities that you/the student demonstrated to compensate for any losses due to the student's vision impairment?	
8. Some papers mention stigma experienced from others in the health professional setting. Was this an issue and how was it managed?	

After the interview each interviewee had the opportunity to read the written transcript and make any changes to ensure the interview content corresponded with their perspectives. Interview content was directly extracted, arranged and reported in a structure aligning with the UK Chartered Society of Physiotherapists framework that provides guidance to educators for supporting disabled students [13] (by study investigator KJ). This analysis was reviewed by the student and clinical educator to ensure their experience and perceptions were accurately reflected, and further written reflections by the student and educator were included at this stage. The physiotherapy program director (SMc), course coordinator (KJ) and disability services manager (SMa) also reviewed the analysis and commented on any other planning, consultation, collaboration and problem solving strategies that contributed toward the clinical placement experience of this student. This evaluation was approved by the University of South Australia Human Research Ethics Committee (Application ID: 0000034593) and written, informed consent was provided by each participant. The student (“Andrew”) has given consent for his case to be published.

## Findings

### Key features of the clinical placement experience

The ten features identified align with the guidance framework published by the Chartered Society of Physiotherapists [13].

1. *Appropriate foundations from program entry to present*: The university policies for implementing the Disability Discrimination Act 1992 meant Andrew’s contact with a disability adviser began before program entry. This adviser remained a consistent point of contact throughout the physiotherapy program. Upon acceptance into the physiotherapy program a Disability Service Access Plan was developed that detailed adjustments and tools to be provided by the disability service including technological devices and modifications to teaching and learning materials and assessments.
2. *Resources including technological assistance to access acute care course content*. Textbooks were available in electronic form. Andrew made extensive use of course content material available as audio recordings or online (so he could use enlarged formats on screen) as well as attending all face-to-face sessions.
3. *Prior learning from other clinical placements*: The order of placement experiences was carefully planned. Andrew’s second year introductory placement included experience in the private practice of a legally blind physiotherapist and working with electronic patient records. In third year, clinical placements managing patients with musculoskeletal conditions in an outpatient setting and orthopaedic and neurological conditions in an inpatient rehabilitation setting were scheduled first. Experience on these placements highlighted Andrew’s strengths and likely challenges in the acute hospital setting. Strengths included his well-developed communication skills, ability to assess and intervene effectively in patients with gait or postural abnormalities, competency in risk assessment and appropriate help-seeking behaviour when indicated. Challenges included requirement for extra space and time to use a portable magnifying device to read medical records, difficulty reading screen-based monitors and need for extra time to organise the physical environment.
4. *Prioritised early placement organisation*. Placement location was chosen by the program director to facilitate geographical access by public transport. The facility head of department was consulted early to gain their cooperation and support for the acute care clinical placement.
5. *Pre*-*placement visit and meeting with clinical educator*. In line with recommendations [12] this meeting included the student, educator and university staff and provided an opportunity to begin familiarisation with the facility environment and discuss planned adjustments and supports.
6. *Making reasonable adjustments*: Recognised as a “cornerstone of disability legislation in relation to promoting equality of opportunity for disabled people” [13, p59] this was the single most important feature identified as essential for placement success. A support worker (a final year physiotherapy student who had completed all clinical courses) was employed to assist Andrew during 60 % (in week 1) to 40 % of placement hours. Specific tasks performed by the support worker were:
  Locating, navigating and reading from patient medical records as directed by Andrew.
  Observation of bedside environment including reading from screen-based monitors and assistance with organising attachments as directed by Andrew.

The support worker was funded by the University through the disability support services and recruited by the program director.

The educator selected patients who appeared co-operative for Andrew’s first few clinical interactions. During the first week of placement the educator observed that Andrew was managing clinical interactions well and this adjustment was removed. Another adjustment was allowing more time for patient interactions especially early in the placement to organise the environment and write in the medical record. The clinical educator estimated that Andrew required approximately 25 % more time in a new patient interaction overall than fully sighted students, mostly due to more time spent in documentation.

7. *Disclosure*: The student chose to fully disclose the presence and impact of his vision impairment and the support strategies put in place to colleagues and patients. At this clinical placement facility the initial contact with patients was made by the educator, to ascertain willingness to be assessed and treated by a student. This also provided an opportunity for the educator to disclose Andrew’s vision impairment and explain the presence of the support worker. No patients declined on the basis of Andrew’s vision impairment. Open disclosure with staff and patients was a positive feature that promoted problem solving by the student and contributed to placement success.
8. *Student qualities and compensations*: Along with making reasonable adjustments, the qualities and preparedness of the student were considered highly essential in making this placement work. These qualities included
  Professionalism and communication: Andrew had already been in the workforce for 14 years (in personal training and fitness instruction) and these skills were well developed.
  Willingness to learn and engage, proactively identify issues and solve problems.
  Memory: After initial orientation Andrew was able to remember and recall geographical locations and ward layouts.
  High level of preparation and knowledge of course content.
  Use of kinaesthetic and tactile cues as early warning signs of patient’s postural instability: This skill along with a fast reaction time enhanced Andrew’s ability to maintain patient safety during transfers and mobilisation.

Personal qualities including professionalism, communication and willingness were identified in an Australian study of 161 allied health clinical educators as more important requirements in preparation for clinical placement than knowledge, understanding or skills [23].

9. *Cooperation of other students and staff*: As the support worker was only employed in the morning, Andrew required the assistance of a student colleague or educator to read notes associated with patient interactions in the afternoons.

10. *Review of adjustments over time*. The need for an assistant to read medical records at Andrew’s direction was unchanging over time. In other clinical placement environments Andrew used a portable magnifier to read notes, but in the acute care environment with limited desk space and where a high degree of therapist mobility was required this was not feasible. After a few days Andrew led the information gathering process by directing the support worker to search in the notes for the data he required. The support worker and student communicated continually to refine the degree of support required over placement. Other adjustments were scaled back after the first week of clinical placement as previously discussed.

### Placement outcomes

Competency based assessment at the end of placement was not modified; with the reasonable supports in place Andrew demonstrated most performance indicators for each competency requirement to a good or excellent standard on the validated assessment tool, the Assessment of Physiotherapy Practice [24]. The clinical placement was a positive experience for the student and Andrew nominated treating patients in the intensive care unit as one of the most rewarding aspects.

The initial concerns of the educator were transformed during the placement:

“Initially, when I heard I was going to be teaching a visually impaired student, I was quite apprehensive about how it would work in such a fast-paced, busy and often high-pressure environment. The more I thought about it, the more barriers/issues came to mind. I think this was largely attributed to the ‘unknown’, having never experienced such a situation prior. I made a conscious decision to remain open-minded and flexible with our program to ensure that reasonable adjustments could be made. Within the first two weeks of this placement, I quickly recognised that with the afore-mentioned supports in place, a visually impaired student is able to perform just as well as (and sometimes better than!) full sighted students. It was a great experience to be involved in and overall was very positively received in the (hospital) environment” (Andrew’s clinical educator).

## Discussion: should inherent requirement statements be reconsidered?

This case report illustrates how legislative and policy initiatives to welcome and support students with disability were implemented in the potentially challenging acute hospital setting with a high degree of success. Andrew’s experience represents an approach to inclusion of students with a disability in physiotherapy education, perhaps in contrast to the implementation of inherent requirement statements by physiotherapy education providers. The case report prompts further debate about the use of inherent requirement statements. Issues to discuss include the apparent lack of involvement of people with disability in their development process; a question about the need for this level of regulation and the mismatch between likely outcomes of inherent requirements and current professional physiotherapy practice.

### Lack of involvement of people with a disability in development of inherent requirement statements

The expression “nothing about us without us” [25] communicates the principle that no policy should be decided by any representative without the direct participation of members the groups affected by that policy. The development of inherent requirement statements has been undertaken without input by the disability sector or practitioners with disabilities [8]. This may be because inherent requirements are not about disability but rather apply to all students and are simply about the nature of the program being offered by the university. In addition, it could be argued that they are not discriminatory because students can be provided with reasonable adjustments to enable them to meet these requirements [17].

However, inherent requirements are about disability. They describe the core aspects of a program which cannot be modified to meet the needs of students with disabilities, and thus are intended to provide certainty to students with disabilities and teaching staff. An example is explicit statements about requirements for sensory abilities, such as visual acuity [16]. This advice to prospective students with vision impairment suggests they cannot reasonably expect to meet this inherent requirement in the absence of some ‘reasonable adjustment’ which miraculously affords them visual acuity. Our experiences suggest that reasonable adjustments are feasible, but this may not be obvious to a prospective student.

A consequence of not including people with disabilities in the development of inherent requirement statements means that the voice of current practitioners and graduates across a range of disciplines with disabilities is missing. The experiences and views about inherent requirements of registered physiotherapists with a range of disabilities that are successfully practicing in Australia and abroad are not present in this work.

### Need for this level of regulation: over-regulation and net benefit

Inherent requirements may be viewed as a case of regulation. The Australian Government Guide to Regulation [26] suggests that an important principle of policy development is that policies are (a) proportionate to the issue and (b) demonstrate a net benefit.

To evaluate inherent requirement statements in the light of this principle we need to consider the size of the problem and whether these statements are an effective strategy to manage the problem. Program retention rates for students with disabilities are similar to the broader student population (76.2 % compared with 78.8 % for all students in 2010) [27] but large-scale evidence of people with disability inadvertently enrolling in courses that they have no reasonable prospect of completing is lacking. Reasons for non-completion in the broader student cohort include financial hardship and illness [27], issues that are not addressed by inherent requirement statements. The use of inherent requirement regulation may thus be an example of using “sledgehammer” policy to crack a “walnut”-sized issue.

### Likely outcomes of inherent requirement statements

Good practice in regulation is characterised by genuine consultation with affected groups and individuals and the development of regulation impact statements [20]. The potential impact of inherent requirement statements requires further consultation and consideration. Using the inherent requirement statement relating to visual acuity for the physiotherapy program at the University of Western Sydney [16] (Table 2) as an example, we pose some points to consider.

**Table 2:** Physiotherapy inherent requirements - visual sensory ability [16]

*“Adequate visual acuity is required to provide safe and effective physiotherapy management. Student demonstrates sufficient visual acuity to perform a range of skills.*	
*Justification of inherent requirement: Sufficient visual acuity to demonstrate the required range of skills, tasks and assessments to maintain consistent, accurate and safe care to self and others. Visual observations, examinations and assessment are fundamental to safe and effective physiotherapy practice*	
*Adjustments must address the need to perform the full range of tasks involved in clinical practice. Any strategies to address the effects of the Vision Impairment must be effective, consistent and not compromise treatment or safety.*	
*Exemplars: Observing and detecting subtle changes in posture, movement and the ability to perform functional activities during assessment and treatment. Safely operating electrotherapy equipment.”*	

It is likely that many readers will interpret this inherent requirement as meaning that a physiotherapist needs to achieve visual acuity in order to practice. It can be assumed that a prospective student with uncorrectable impaired visual acuity will read the statement as indicating their exclusion from the program of study and practice. This is despite the fact that students such as Andrew continue to successfully complete physiotherapy programs and disregards the past and present cohort of physiotherapists who are registered and practising nationally and internationally.

Based on existing legislation we propose an alternative approach that has been adopted in the case of Andrew and others. In this approach,

- Universities describe their programs clearly and indicate the activities and assessment processes involved.
- Universities advise students with disabilities that reasonable adjustments will be provided to promote access and participation. Universities advise that reasonable adjustments cannot undermine the core requirements of the program.
- The process of determining reasonable adjustments is an individualised one, taking into account the views of the student, professional consultation if required and a balance of interests [28].
- If there are concerns about a student’s capacity to safely undertake practice as a result of their disability, Australian Health Professional Regulation Authority [29] sets out a responsibility for universities to report these concerns. This regulatory body has the legal authority to make a decision regarding the student’s fitness for student registration and thus participation in the program.

This process removes the likelihood of students being self-selected out of programs based on inherent requirement statements that do not carry legal authority and remove the opportunity for the student to negotiate adjustments. As demonstrated by the experience of Andrew, his educator and his patients during clinical placement in the acute hospital setting, outcomes achieved with the provision of reasonable adjustments may not be able to be predicted from the starting point of abstract inherent requirement statements.

## Summary

Using a case report and policy discussion this paper highlights the exciting potential of inclusive practice. Planning, consultation, collaboration and problem solving led to a successful individualised approach to making reasonable adjustments for a student with a disability to be highly successful in an acute hospital setting. The implementation of inherent requirement statements may systemically reduce the capacity of education providers to develop bespoke solutions in response to individual circumstances. This may be a barrier to developing competent, diverse and creative health practitioners, with representation of people with disabilities and building a workforce to meet the challenges of the future.

## Abbreviations

OECD: Organisation for Economic Cooperation and Development
UK: United Kingdom
